# Barriers to and facilitators of online health information-seeking behaviours among cancer patients: A systematic review

**DOI:** 10.1177/20552076231210663

**Published:** 2023-12-15

**Authors:** Giulia Ferraris, Dario Monzani, Veronica Coppini, Lorenzo Conti, Silvia Francesca Maria Pizzoli, Roberto Grasso, Gabriella Pravettoni

**Affiliations:** 1 Applied Research Division for Cognitive and Psychological Science, IEO, European Institute of Oncology IRCCS, Milan, Italy; 2 Department of Psychology, Educational Science and Human Movement (SPPEFF), University of Palermo, Palermo, Italy; 3 Department of Oncology and Hemato-Oncology, University of Milan, Milan, Italy; 4 Faculty of Psychology, Psychology Department, Università Cattolica del Sacro Cuore, Milan, Italy

**Keywords:** Online health information-seeking behaviours, cancer, barrier, facilitator, digital divide, Internet

## Abstract

**Objective:** Disparities in cancer care contribute to higher rates of cancer mortality. Online health information would be a resource for cancer patients to obtain knowledge and make health decisions. However, factors that hinder or facilitate online searching behaviours among patients remain unexplored. The current systematic review aims to identify and synthesise evidence of cancer patients’ barriers to and facilitators of online health information-seeking behaviours. **Methods:** Electronic databases (PubMed, EMBASE, Scopus) were systematically searched, and a total of 123 full-text studies were reviewed of which 24 met the inclusion criteria. **Results:** Thematic analysis was performed to identify barriers and facilitators of online health information-seeking behaviours. Seven key themes were identified: (1) socio-demographic characteristics (age, gender, education, income, ethnicity and language), (2) psychosocial aspects (psychological wellbeing, need for a face to face contact, motivation, support), (3) accessibility (Internet access, residence), (4) quality and quantity of information (amount, reliability), (5) cancer stage and symptoms (time since diagnosis, experiencing symptoms), (6) aspects related to healthcare professionals (relationship with the patients and opinions on online health information) and (7) digital literacy (computer skills and literacy). **Conclusions:** Findings underscore the significance of recognising the multifaceted nature of barriers and facilitators affecting cancer patients’ online health information-seeking behaviours. A strong link between these factors and cancer patients’ ability to make informed decisions and cope effectively with their diagnosis emerged. Consequently, addressing these barriers and leveraging the identified facilitators could lead to improvements in patient-centred care, ultimately contributing to better healthcare services and informed decision-making for cancer patients. Future research should prioritise exploring strategies for enhancing cancer care accessibility across all stakeholders involved.

**Registration:**

CRD42023408091

Disparities in cancer care contribute to higher cancer mortality rates.^1^ Online information can be a valuable resource to guide cancer patients worldwide and a first step towards providing better and broader access to cancer healthcare services. However, factors hindering or facilitating online information-seeking behaviours among cancer patients produce inconsistent results and remain questionable. Therefore, the current systematic review is aimed at gaining a greater understanding of cancer patients’ barriers to and facilitators of online health information-seeking behaviours.

In recent years, the interest in the use of the Internet as a means of delivery of online health information has rapidly grown in the general population^2^ as well as in the cancer context.^3^ Findings from the first Health Information National Trends Survey (HINTS) indicated that cancer patients often search for online cancer-related information before talking with their healthcare professionals.^4^ Reasons for searching online information span from the need to understand and manage disease symptoms, to finding the best treatment options, and preparing for patient-doctor communication.^5^ Generally, searching for information online produces advantages for cancer patients. For example, online information has enormous potential to improve individuals’ health by overcoming barriers of time and space. Moreover, positive attitudes towards searching the Internet for health information often make cancer patients informed and actively involved in the decision-making process, which are all key factors for person-centred care.^6^ However, online information-seeking behaviours are not always present and they differ according to personal, socio-demographic and contextual factors, determining disparities and inequalities in accessing information.^7^ For instance, online health information seekers tend to be better educated and wealthier than those who do not seek online health information.^8^ Cognitive abilities, such as information processing skills and health literacy, are also considered major facilitators of online information seeking.^9^ Moreover, the use of the Internet for online health information seeking is greater among Caucasians than among ethnic minorities^10^ and among the urban population than among people living in rural areas.^11,12^ Also, not being employed full-time, having good digital literacy and being female have been identified as consistent influences of higher Internet health information searching.^13^ On the other hand, other studies focussing on cancer information have found mixed results on the influence that demographic characteristics such as age, gender, education and income can have on online information seeking.^14^ For example, younger patients were found to seek more online health information than older ones^15,16^ whereas, in other studies, older adults were the ones seeking more information online^13^ or no correlation was found between age and online information seeking.^17^ Moreover, there is a wide range of psychosocial factors that produced controversial findings. For instance, psychological distress was found to lead to either apprehension or avoidance of information, specifically cancer-related anxiety and beliefs have been found to be associated with decreased online information-seeking behaviours^18^ as well as greater information-seeking behaviour.^19^ Lastly, patients may look for online information because they are not satisfied with what is reported by their healthcare professionals; however, there are cases when patients look for online information to complement what they have been told by their healthcare professionals.^20^

Therefore, there is a need to systematise existing literature in order to highlight where there is room for further investigations and clarify in which situations results are mixed in terms of barriers to and facilitators of online cancer information-seeking behaviours. Identifying barriers and facilitators is important for a number of additional reasons. First, it is widely accepted that patients have better healthcare outcomes when they are more informed about their disease, further involved with their treatment choices and more invested in their healthcare pathways. Therefore, online informative materials can produce positive outcomes for cancer patients in terms of a better quality of care, but also in terms of prevention.^14^ Secondly, understanding the factors associated with health-related Internet use would be useful in designing strategies aimed at reducing the digital divide. The digital divide is defined as the gap between people who have access to technology and those who do not have access to it.^21^ Many factors might contribute in reducing the digital divide, including socio-demographic access (e.g., age, education, race/ethnicity, residence and health outcome)^22^ as well as psychological responses (e.g., trust and self-efficacy) and physical access to technologies (e.g., Internet access, eHealth literacy).^23^ Thirdly, broader knowledge of which factors facilitate or, on the contrary, hinder the use of the Internet for cancer-related information, might inform web-based education and digital information tools as well as guide the development of innovative and personalised eHealth interventions for cancer patients.^24,25^

In line with the Comprehensive Model of Information Seeking (CMIS^14^) the current systematic review aims to identify, apprise and synthesise evidence of patients’ barriers to and facilitators of online health-related information-seeking behaviour among cancer patients to shed light on the potential disparities in accessing online health-related information and support. The CMIS was developed to investigate the predictors of health information-seeking behaviour based on the characteristics and perceptions of information seekers; the model was originally developed for information in a traditional media context; however, the current systematic review refers to an expansion of the CMIS advanced by van Stee & Yang (2017)^14^ which considers the variable of the online/Internet use.

## Methods

The guidelines of the Preferred Reporting Items for a Systematic Review and Meta-analysis (PRISMA^26^; see Supplementary Material 1) were followed in conducting the current review. Moreover, the protocol of the present systematic review was registered in the PROSPERO international Prospective Register of Systematic Reviews database in advance of the review being conducted (registration ID = CRD42023408091). Finally, the PICOS tool was implemented to highlight the aim of the current review, although it was not feasible to include the comparison element due to the objective of this review^27^ (see supplementary material 2).

### Eligibility criteria

#### Participants

The population comprehended adult cancer patients at any stage of the disease or cancer survivors. The studies that counted only minors (<18 years old), healthy subjects, patients’ relatives, informal caregivers or healthcare professionals in the sample were excluded; mixed sample studies were also excluded.

#### Phenomenon of interest

The phenomenon of interest of the present review is online cancer information-seeking behaviours and health-related Internet search among cancer patients or cancer survivors. The main focus was on the behaviour, not on the source of the search, thus, studies with evaluations of web pages or apps were excluded. The studies that targeted, mentioned or evaluated offline sources of information seeking, such as pamphlets or television, and telemedicine were excluded too. Any other type of digital intervention was excluded as well in order to better observe the factors influencing the active behaviour of online information seeking. Finally, where Internet use was related to only seeking support, not information, and social media support groups, studies were excluded.

##### Outcome

The studies were included when the main outcome provided information on barriers to and facilitators of the online information-seeking behaviours in cancer patients or survivors. Barriers and facilitators suitable for inclusion consisted of socio-demographic characteristics, illness-related characteristics, lifestyle, psychological factors, but also external factors such as culture, social norms, financial factors, broader social environment and interpersonal relationships.

##### Study design and publication type

Studies with qualitative, quantitative and mixed methods designs were included. Single-case studies were not included due to lack of generalisability. The current systematic review included all the studies that were published in the English or Italian language. Reviews, protocols, conference abstracts, editorials and other types of publication considered as grey literature were excluded.

### Information source and search strategy

The search sources and online databases used to identify the studies for the present systematic review were PubMed, Scopus and EMBASE. No restrictions were applied regarding the year of publication, and the search was conducted from database inception to March 2023. The search strategy was developed in consultation with a librarian at the European Institute of Oncology. The search strategy was designed in PubMed and then translated to the appropriate MESH/thesaurus terms and formats for the other databases. The literature search was constructed based on terms related to the following PICOS criteria: (1) cancer patients or survivors (e.g., neoplasms, cancers, tumours, oncology, malignant, metastasis), (2) online cancer information-seeking behaviours (e.g., information-seeking behaviour, health information exchange, access to information, health literacy, Internet access, Internet use, online, social media, web) and (3) barriers and facilitators contributing to information-seeking behaviours (e.g., digital gaps, attitude to health, health beliefs, fear, anxiety, shame, social support system, emotional wellbeing, psychosocial factors). The complete search strategy is detailed in supplementary material 2. Apart from conducting the initial search, we also performed backward and forward reference searches on the included studies. However, these additional searches did not yield any new studies beyond what was already retrieved through the database search.

### Study selection process

The studies retrieved from the search were uploaded to the Rayyan software^28^ in order to facilitate the study selection process. The Rayyan software performed an initial identification of duplicates, which was then checked manually. Next, the studies were sorted into alphabetical order by the author's name to facilitate independent screening of all records by the reviewers (GF, VC, LC). Each reviewer evaluated the eligibility of the studies by title and abstract, reporting decisions to Rayyan in the blind-on mode to avoid influencing the evaluation of the other authors during the Inter-Observer Agreement (IOA) assessment phase.^29^ In order to ensure greater reliability in the selection of the studies, those evaluated by each reviewer (e.g., GF or VC) were independently re-evaluated by another reviewer (LC or SP). The percentage of IOA was 85%. After switching to blind-off mode, conflicts, if present, were resolved by discussion, and a third reviewer (DM) was consulted as needed. The reconciliation process was performed after calculating the percentage of agreement.^29^

### Data extraction

All data were extracted by one reviewer (VC) and confirmed to be accurate and complete by another reviewer (GF, LC, or DM, or SP) in the same Excel sheet. The authors discussed with each other daily in order to ensure a coherent data collection methodology and accurate data extraction. Data from the included studies related to country, study design, objectives of the study, sample size, age range, mean and standard deviations, ethnicity, diagnosis and data collection type were extracted using an Excel sheet (version 2016; Microsoft Corporation), as reported in Table 1. The scope of Internet use was collected, separately as reported in Table 2. Barriers and facilitators were organised in Table 3.

**Table 1.** Results of included studies.

**ID**	Author	Country	Study design	Objective	Sample size	Age range, and or Mean (SD)	Gender %	Ethnicity %	Diagnosis	Data collection type
1	An et al. (2016)	USA	Cross-sectional	Understand cancer patients’ use of the Internet as an informational resource	N = 1282	54% of subjects 50-69, **—**	F = 59%	White 88%	Leukemia/lymphoma Breast cancer	Paper-based Survey
2	Balka et al. (2010)	Canada	Qualitative	Utilisation of the Internet as a means of health information consumption amongst young women with breast cancer	N = 35	28-45, 39 (**-**)	F = 100%	**—**	Breast cancer	Written Narratives
3	Bender et al. (2019)	Canada	Cross-sectional	Determine the patterns and factors associated with the use of the Internet as a source of health information	N = 1362	43-95, 69 (8.2)	M = 100%	**—**	Prostate cancer	Online Survey
4	Casellas-Grau et al. (2018)	Spain	Cross-sectional	Analyse how post-traumatic stress and growth are related to the amount of time spent looking for online information and the type of content	N = 182	21-73, 46. (8.2)	F = 100%	**—**	Breast cancer (survivors)	Questionnaire
5	Chou et al. (2011)	USA	Longitudinal	How cancer survivors in the USA utilise the Internet for health-related purposes	N = 2637	37% of subjects >65, **—**	F = 59%	Non-Hispanic white 86%	Cancer survivors	Online Survey
6	Corrales et al. (2018)	USA	Cross-sectional	Describe the use of the Internet for health information research and examine the association between its use and anxiety	N = 212	18-90, 57.8 (15.3)	F = 100%	White 57%	Gynecologic cancer	Online Survey
7	Dickerson et al. (2011)	USA	Qualitative	Understand the experience of men with cancer using the Internet	N = 15	47-78, 63.3 (10)	M = 100%	**—**	Prostate cancer leukemia	Interview
8	Duimel et al. (2022)	The Netherlands	Cross-sectional	Identify theoretically-founded profiles of cancer patients differing in their motives for seeking informational and/or emotional support online	N = 211	20-88, —	F = 51.9%	**—**	Breast cancer Urological cancer Cancer survivors	Questionnaire
9	Fleisher et al. (2002)	USA	Cross-sectional	Examine the relationship between use of Internet health information by people newly diagnosed with cancer, patient task behaviour and perceived self-efficacy	N = 313	41% of subjects 41-70, **—**	**—**	White 90%	Prostate cancer Breast cancer	Interview
10	George et al. (2018)	USA	Multivariable modeling	Examine patterns, correlates, and the impact of cancer-related Internet use among patients with advanced cancer	N = 180	50% of subjects ≤ 60, **—**	F = 52%	Non-Hispanic white 82%	Colorectal cancer Lung cancer Gynecologic cancer Head and neck cancer Breast cancer Melanoma	Questionnaire
11	Haase et al. (2020)	Canada	Qualitative	Examine the use of the Internet for cancer information amongst older adults with cancer	N = 17	**—**, 65.5 (0.9)	F = 58%	**—**	Breast cancer Prostate cancer Colorectal cancer	Interview
12	Jiang and Liu (2019)	USA	Longitudinal	Explore the trend of Internet health information seeking (IHIS) in cancer survivors and the relationship between four dimensions of digital divide and IHIS	N = 1526	**—**, 65.7 (-)	F = 61%	Non-Hispanic white 76.4%	Breast cancer Prostate cancer	Online Survey
13	Kowalski et al. (2014)	Germany	Longitudinal	Look into correlations between Internet utilisation and sociodemographic characteristics and if these change over time	N = 27491	**—**, 61.1 (-)	**—**	**—**	Breast cancer	Questionnaire
14	Mattsson et al. (2017)	Sweden	Cross-sectional	Investigate health-related Internet use among people with cancer	N = 282	20-90, 65.2 (-)	M = 53%	**—**	Prostate cancer Breast cancer Lymphoma	Questionnaire
15	Mayer et al. (2007)	USA	Cross-sectional	Examine the cancer information-seeking behaviours and preferences of cancer survivors	N = 619	**—**, 60 (-)	F = 64%	Caucasian 79%	Breast cancer Cervical cancer Prostate cancer Melanoma Colorectal cancer Endometrial cancer (all survivors)	Interview
16	Melhem et al. (2023)	Jordan	Cross-sectional	Explore cancer survivors’ information-seeking behaviour, information sources, digital health literacy, and digital trends	N = 335	35% of subjects 50-59, (-)	F = 83%	**—**	Breast cancer Colorectal cancer (all survivors)	Online Survey
17	Paul et al. (2011)	Australia	Cross-sectional	Assess the reported level of access to the Internet, preferred sources of information, and preferred sources of support among survivors of hematologic cancers	N = 268	**—**, 59.5 (13.4)	M = 57%	**—**	Leukemia Lymphoma Myeloma Lymphoma	Paper-based Survey
18	Peterson and Fretz (2003)	USA	Cross-sectional	Determine how frequently patients attending a lung cancer clinic use the Internet for their own health information and how patients compare the quality of Internet information with other sources of lung cancer information	N = 139	**—**, 58.5 (15.4)	M = 61%	**—**	Lung cancer	Questionnaire
19	Rising et al. (2015)	USA/ Netherlands	Cross-sectional	Explore use and perceptions about eHealth among men living with prostate cancer	N = 289	40-89, 64.9 (8.3)	M = 100%	White 95%	Prostate cancer	Questionnaire
20	Thomson et al. (2012)	USA	Cross-sectional	Explore the characteristics of colorectal cancer patients who accessed Internet-based health information as part of their symptom appraisal process prior to consulting a health care provider	N = 242	**—**, 56.5 (-)	F = 50%	African American 44%	Colorectal cancer	Interview
21	Tian and Robinson (2008)	USA	Longitudinal	Compare the health information and media usage patterns of older adults diagnosed with cancer with their younger adult counterparts	N = 401	65% of subjects <65, 61.7 (-)	F = 63%	Caucasian 91%	Various types of cancer	Online Survey
22	Valero-Aguilera et al. (2012)	Spain	Cross-sectional	Describe the profile of urological cancer patients who look for health information on the Internet and analyse the factors related to use of the Internet as a source of health information	N = 169	**—**, 72.2 (-)	M = 95%	**—**	Urological cancer	Interview
23	Valero-Aguilera et al. (2014)	Spain	Cross-sectional	Describe the information needs of urological and breast cancer patients and factors related to use of the Internet as a source of health information	N = 269	**—**, 54.8 (-)	**—**	**—**	Urological cancer Breast cancer	Interview
24	Yli Uotila et al. (2013)	Finland	Qualitative	Describe why Finnish cancer patients choose the Internet as a source of social support	N = 74	24-72, 53 (-)	F = 87%	**—**	Breast cancer Gynecological cancer	Questionnaire

**Table 2.** Scope of Internet use in the included studie*s*.

		Study ID
Scope of Internet use	General Health and Cancer related information	1; 2; 3; 4; 5; 6; 11; 12; 13; 14; 15; 16; 17; 18; 21; 22; 24
	Treatment options and implications	1; 2; 3; 6; 7; 9; 10; 11; 19; 20; 22; 23
	Coping modalities, emotional support and other patients’ experience	1; 2; 4; 8; 19; 24
	Prognosis and likelihood of survival	2; 6; 7; 19; 23
	Clinics, hospitals and best medical experts	1; 6; 7; 14
	Schedule appointments	10; 14
	Buy Medicines	12

*Note:* The Study ID numbers refer to the ID of the included studies reported in Table 1.

**Table 3.** Themes and subthemes as barriers and facilitators in the included studies.

Socio-demographic characteristics	Psychosocial aspects	Accessibility	Quality and quantity of information	Cancer stage and symptoms	Aspects related to healthcare professionals	Digital literacy
**Barriers**						
Older age [ID: 1; 5; 10; 12; 13; 14; 17; 18; 19; 20; 21; 22; 23]	Feeling of anxiety, confusion and overwhelmedness [ID: 4; 10; 19]	Living in rural areas [ID: 5; 22; 23]	Complexity, stressfulness and reliability of the information [ID: 2; 3; 7; 9; 15; 16; 18]	Higher cancer stage [ID: 5; 13; 19]	Preference for consulting a healthcare professional [ID: 6]	Limited computer skills/low digital literacy [ID: 16]
Lower education [ID: 1; 5; 12; 14; 17; 23]	Post traumatic stress related symptoms [ID: 4]	No or limited access to the Internet [ID: 6; 8]		Not having symptoms [ID: 20]	Being discouraged from using the Internet from a healthcare professional [ID: 11]	
Lower income [ID: 10; 12; 15; 18; 23]	Need for face to face contact [ID: 2]					
Ethnic minority and/or foreign native language [ID: 5; 12; 13; 16]	The notion of unnecessariness [ID: 6; 15]					
Male gender [ID: 1; 17]	Lack of interest and motivation [ID: 5; 6]					
Unemployment [ID: 23]	Living alone and/or having no friends and family and/or being unmarried [ID: 13; 14; 17]					
Not having insurance coverage [ID: 20]						
**Facilitators**						
Younger age [ID: 1; 3; 6; 10; 12; 13; 14; 15; 16; 17; 19; 20; 21; 23]	Confidence in searching for information, self-efficacy and no psychological distress [ID: 3; 5; 7; 9; 12; 19; 23]	Living in urban areas [ID: 3; 5; 22; 23]	Perception that the Internet was useful for social support and health related information [ID: 1; 7; 15; 19]	Having just been diagnosed [ID: 5; 6; 9; 14]	Concern with knowledge limitation of physician, not having access to a healthcare professional [ID: 7; 8; 11; 24]	Good computer skills and digital literacy [ID: 3; 8]
**Facilitators**						
Higher education [ID: 1; 3; 6; 8; 12; 13; 14; 16; 17; 18; 20; 22; 23]	Need for control, emotional/informational needs, anxiety and negative feelings [ID: 2; 3; 6; 7; 14; 24]	Broadband Internet access, multiple devices [ID: 3; 12; 15; 24]		Having a comorbid condition or undergoing an aggressive treatment [ID: 16; 23]	Having a regular healthcare provider or other family members that search for information online and good patient task behaviour [ID: 6; 9; 15]	
Higher income [ID: 3; 6; 10; 12; 15; 16; 18; 20; 22; 23]	Experiencing embarrassing symptoms [ID: 20]				Being referred to trusted websites from clinicians [ID: 11]	
Having insurance coverage [ID: 5; 13; 20]	Post traumatic stress symptoms and growth [ID: 4]					
Employment [ID: 16; 17; 23]	Being married, having family and friends [ID: 6; 9; 13; 14; 17; 22]					
Ethnic majority, country of residence language native speakers [ID: 5; 12]						
Female gender [ID: 1; 15]						

*Note:* ID numbers refer to the ID of the included studies reported in Table 1 and in the Results section.

### Quality assessment

The methodological quality assessment of the included studies was evaluated using the Mixed Methods Appraisal Tool (MMAT^30^) suitable for assessing the quality of different categories of studies. Specifically, for the current systematic review, the quality of qualitative and quantitative studies was assessed. For each method five closed questions regarding the approach, the sampling, the data collection and the analysis and interpretation methods were answered by two independent reviewers (GF and VC) that assigned zero point if the answer was ‘no’ and one point if the answer was ‘yes’, as prescribed in the MMAT guidelines (Table 4). Discrepancies were discussed, and consensus was reached.

**Table 4.** Quality assessment of the included studies following the criteria from the mixed methods appraisal tool.

**Included studies**	**Qualitative studies**					**Quantitative studies**					**Total quality score**
	**1.1**	**1.2**	**1.3**	**1.4**	**1.5**	**4.1**	**4.2**	**4.3**	**4.4**	**4.5**	
An et al. (2016)						1	1	1	0	1	4/5
Balka et al. (2010)	1	1	1	1	1						5/5
Bender et al. (2019)						1	1	1	0	1	4/5
Casellas-Grau et al. (2018)						1	1	1	1	1	5/5
Chou et al. (2011)						1	1	1	1	1	5/5
Corrales et al. (2018)						1	1	1	1	1	5/5
Dickerson et al. (2011)	1	1	1	1	1						5/5
Duimel et al. (2022)						1	1	1	0	1	4/5
Fleisher et al. (2002)						1	1	1	0	1	4/5
George et al. (2018)						1	1	1	0	1	4/5
Haase et al. (2020)	1	1	1	1	1						5/5
Jiang et al. (2019)						1	1	1	1	1	5/5
Kowalski et al. (2014)						1	1	1	1	1	5/5
Mattsson et al. (2017)						1	1	1	0	1	4/5
Mayer et al. (2017)						1	1	1	1	1	5/5
Melhem et al. (2023)						1	1	1	0	1	4/5
Paul et al. (2011)						1	1	1	0	1	4/5
Peterson et al. (2003)						1	1	1	0	0	3/5
Rising et al. (2015)						1	1	1	1	1	5/5
Thomson et al. (2012)						1	1	1	0	1	4/5
Tian et al. (2008)						1	1	1	0	1	4/5
Valero-Aguilera et al. (2012)						1	1	1	0	1	4/5
Valero-Aguilera et al. (2014)						1	1	1	0	1	4/5
Yli Uotila et al. (2013)	1	1	1	1	1						5/5

*Note*: 0 = No; Cannot tell; 1 = Yes; Qualitative studies: 1.1. Is the qualitative approach appropriate to answer the research question? 1.2. Are the qualitative data collection methods adequate to address the research question? 1.3. Are the findings adequately derived from the data? 1.4. Is the interpretation of results sufficiently substantiated by data? 1.5. Is there coherence between qualitative data sources, collection, analysis and interpretation? Quantitative studies: 4.1. Is the sampling strategy relevant to address the research question? 4.2. Is the sample representative of the target population? 4.3. Are the measurements appropriate? 4.4. Is the risk of nonresponse bias low? 4.5. Is statistical analysis appropriate to answer the research question?

### Data synthesis

The thematic analysis of data was conducted to identify and synthesise themes of barriers to and facilitators of health-related online information-seeking behaviours.^31^ Data related to information-seeking behaviours were primarily deductively coded referring to the CMIS.^14^ However, data that did not fit within the above-mentioned model were inductively coded. Qualitative data on barriers and facilitators were integrated by creating narrative summaries and reported in an Excel spreadsheet. Quantitative data extracted from quantitative studies underwent a conversion process, turning them into ‘qualitatively described data’ for seamless integration with qualitative information.^32^ This step involved translating numerical findings into textual narratives and interpretative descriptions. Subsequently, a thematic analysis was executed as a means of integration. During this stage, the ‘qualitatively described’ data were amalgamated and merged with the outcomes from qualitative studies to identify common barriers and facilitators based on similarity.

Included studies were independently coded by two reviewers (GF and VC). This was followed by a discussion between more reviewers (GF, VC, DM, LC, SP, RG, GP) to reach a shared understanding of the themes. Similar barriers or facilitators were grouped together in descriptive sub-themes in order to avoid repetition and overlapping (e.g., finding the amount of information overwhelming was associated with finding the amount of information to be stressful or confusing^33,34^). As reported in Table 3, themes were listed with reference to each article in which the barrier or facilitator was detected, this allowed the authors to assess the frequency of each subcategory and, theme permitting, a more accurate analysis and interpretation of the results.

## Results

### Study selection

**Figure 1.**
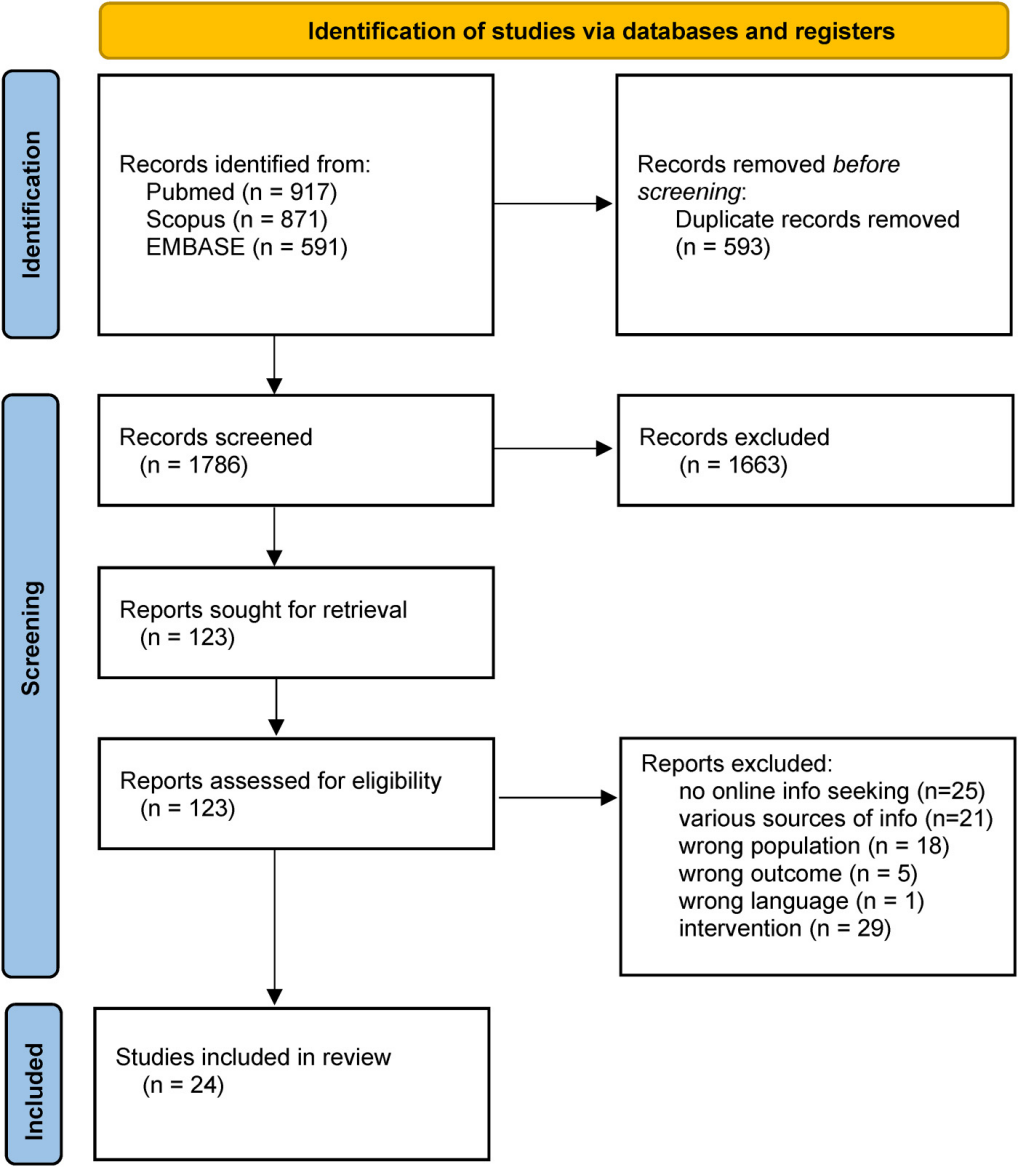
PRISMA 2020 flow diagram for new systematic reviews which included searches of databases and registers only.

The search from the databases PubMed, Scopus and EMBASE resulted in 2379 studies. Duplicates (n = 593) were removed and a total of 1786 studies were screened by title and abstract. Out of these, 1663 were subsequently excluded; at last, the full-text analysis of the remaining 123 studies was conducted. Out of these, 99 were excluded, as they did not meet the eligibility criteria. A total of 24 studies were finally included. Figure 1 shows the flow chart of the selection process in detail, together with the reasons for exclusion.

### Quality of the studies

The majority of the studies obtained four (n = 12) and five (n = 11) points out of five points and only one study gained three points. The quality assessment results for each study are reported in Table 4.

### Study characteristics

**Figure 2.**
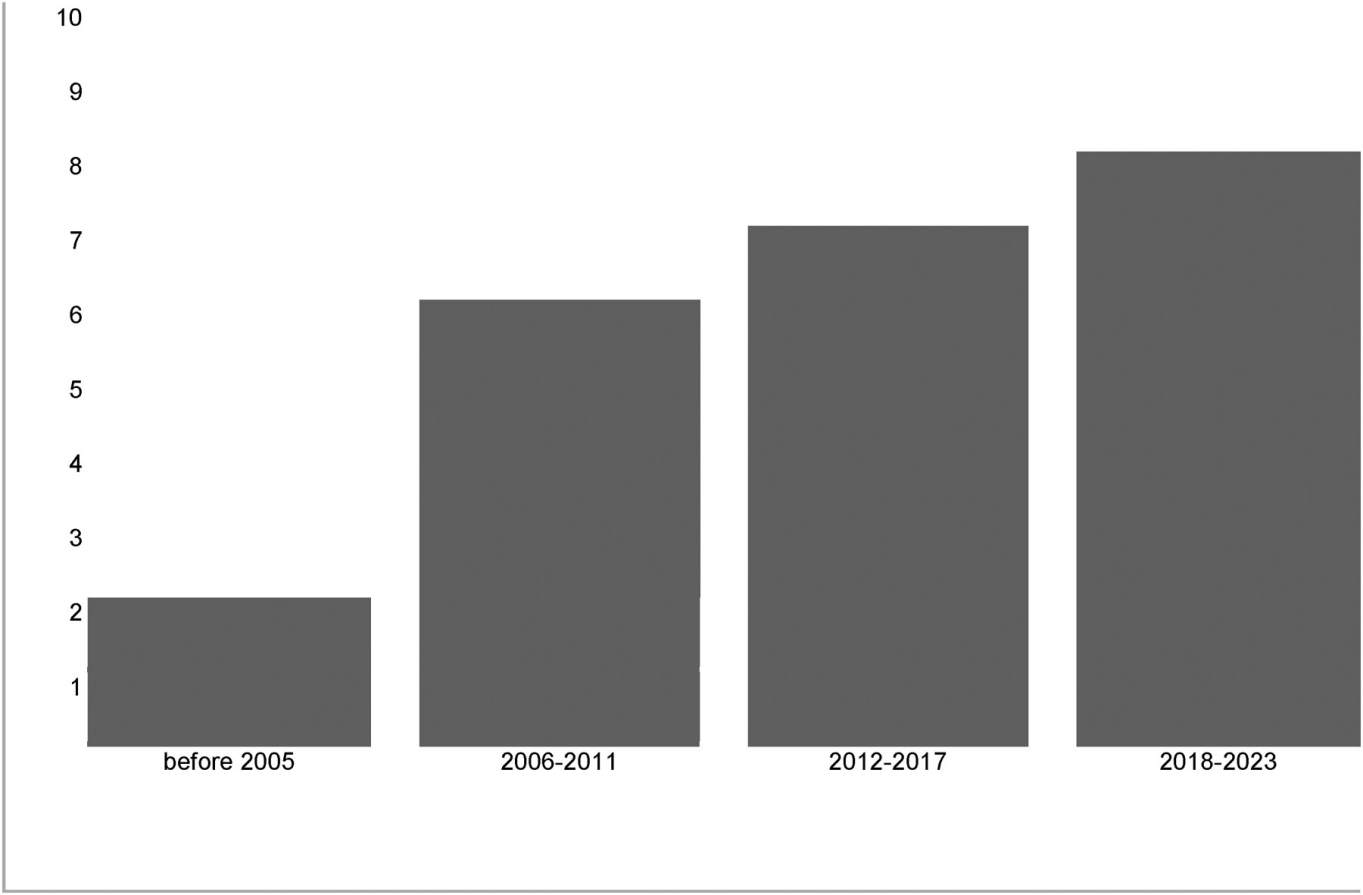
Publication years distribution of the included studies.

The studies included in the current review cover the last 20 years, being published between 2002 and 2023 (Figure 2); most of the studies (62%) were carried out in the USA and Canada,^22,33–46^ just two of them were performed in Jordan and Australia,^47,48^ and the remaining 33% were conducted across different European countries.^24,44,49–54^ The preponderance of the studies (n = 20)^22,24,34–36,38,39,41–53^ was quantitative (16 cross-sectional, 4 longitudinal), and the others were qualitative (n = 4).^33,37,40,54^ The sample sizes of the studies ranged from 15^37^ participants to 27,491.^22,50^ The age of the participants ranged from 18 to 95 years, but most of the studies indicated, approximately, a mean age of 59. The most common diagnosis among the included studies was breast cancer (58%),^24,33,35,38,40–42,47,50,51,53,54^ followed by urological cancer (predominantly prostate cancer; 45%)^24,34,37,38,40–42,44,51–53^ and colorectal cancer (20%).^39,40,42,45,47^ Further information of the study characteristics can be found in Table 1.

The scope of Internet use was predominantly to obtain information regarding cancer and health in general (70% of the studies),^22,33–36,40–43,46–52,54^ followed by the search for the best treatment options and the subsequent implications (50%)^33–40,44–46,53^ and for the coping mechanisms and experiences of other patients (25%).^24,33,35,44,49,54^ Information regarding prognosis and likelihood of survival was researched by cancer patients participating in five studies^33,36,37,44,53^; clinics, hospitals and best medical experts were sought by the participants of four studies^35–37,51^ and, lastly, the Internet was used in order to buy medicines and schedule appointments^39,41,51^ (see Table 2).

### Synthesis of results

In the current review seven different themes of barriers and facilitators were established: socio-demographic characteristics (mentioned in 75% of the studies), psychosocial aspects (75%), accessibility (37%), quality and quantity of information (37%), cancer stage and symptoms (37%), aspects related to healthcare professionals (29%), and digital literacy (12%).

#### Barriers

*Socio-demographic characteristics.* Socio-demographic characteristics were the most recurrent barriers, being reported in 62% of the included studies. Older age was (approximately above 59) the most frequent barrier when it comes to Internet use and online information-seeking behaviour.^22,35,39,41–46,48,50–53^ Lower education and lower income, respectively addressed in six^22,35,41,48,51,53^ and five^39,41–43,53^ studies, have also been evaluated as relevant barriers. For example, Jiang and colleagues (2019),^41^ noted how less than 8 years of institutional education and income between 0 and $9999 a year were strongly related to less frequent online information-seeking behaviours. The absence or low frequency of online information-seeking behaviour was occasionally associated with belonging to an ethnic minority group (e.g., Hispanic in the USA^22^) and speaking a foreign language (e.g., other than German in Germany^50^), as reported in four studies.^22,41,47,50^ At last, male gender,^35,48^ being unemployed^53^ and not having insurance coverage^45^ were also mentioned as possible barriers in cancer patients.

*Psychosocial aspects.* Feelings of anxiety, confusion and overwhelmedness led to avoidance and discouraged the participants from seeking online information.^39,44,49^ Post-traumatic stress-related symptoms (e.g., re-experiencing, avoidance/numbing and hyperarousal following the diagnosis) were also detected as barriers influencing patients’ psychological wellbeing. Moreover, patients with post-traumatic stress symptoms were found to avoid seeking any type of information due to high levels of perceived distress while looking for online information.^33^ Another barrier to online information seeking was found in breast cancer patients who reported the need for face-to-face contact with friends, family and doctors, instead of the consultation of online sources.^33^ Another psychosocial barrier was the perception that searching for online information is not necessary or essential. Often patients stated not having time for it or not being interested or motivated.^22,36,42^ Lastly, lack of a social support system, as being unmarried and not having any friends or relatives, also played a role in three studies.^48,50,51^

*Accessibility.* In three studies,^22,52,53^ cancer patients living in rural areas were less likely to search for information on the Internet in comparison to those living in other areas, especially for health information search purposes. Having no or limited access to a computer or the web was found to be a barrier as cancer patients could not access the Internet and, hence, information.^24,36^

*Quality and quantity of information.* Participants of the included studies addressed the complex matter of the legitimacy and reliability of the health-related information that one can find online as potential barriers to online information seeking.^33,34,37,38,42,43,47^ Cancer patients were overwhelmed, stressed, anxious and depressed, not only by the quantity and the complexity of the information available, but also by the fact that often online information conflicted with one another and was not tailored to the individual. For example, Bender and colleagues (2019)^34^ reported that some patients were confused on how and what information applied to them, others reported having difficulties in finding clear and understandable information.

*Cancer stage and symptoms.* In relation to patients’ health conditions, three studies reported higher cancer stage being associated with the absence, or lower frequency, of online information-seeking behaviours.^22,44,50^ However, not perceiving any symptoms, was also associated with lower frequency of searching for information online in colorectal cancer patients.^45^

*Aspects related to healthcare professionals.* Barriers to online-seeking behaviours were found in cancer patients who expressed their preference for consulting directly with healthcare professionals instead of searching for cancer-related information online.^36^ Whilst in another included study patients with various types of cancer reported being discouraged by healthcare professionals themselves who referred to not looking up anything under ‘Dr Google’ because it ‘gets one's mind going’.^40^

*Digital literacy.* A connection between lower digital and eHealth literacy, together with limited computer skills, was found to be a strong barrier to online information seeking. Indeed, breast and colorectal cancer survivors with low or none digital and eHealth literacy were found to be less likely to acquire information online and to receive technology-enabled cancer care.^47^

#### Facilitators

*Socio-demographic characteristics.* Similarly to the identified barriers, age played a crucial role also as a facilitator. Indeed, younger age was mentioned as a facilitator of online information seeking in 15 studies.^24,34–36,39,41,42,44–48,50,51,53^ Comparably higher education and greater income also seemed to be facilitators, being reported, respectively, in 13^24,34–36,41,43,45,47,48,50,51–53^ and 10 studies^34,36,39,41–43,45.,47,52,53^ Moreover, having insurance coverage^22,45,50^ and being employed,^47,48,53^ again, showed to be more cited as facilitators. Belonging to an ethnic majority and being native speakers in the country of residence was not as present as a facilitator the way it was as a barrier; however, it contributed to online information seeking of the participants of two studies.^22,41^ Lastly, two studies mentioned the female gender as a facilitator of online information seeking in patients with various types of cancer and cancer survivors as well.^35,42^

*Psychosocial aspects.* For cancer patients, self-efficacy and low psychological distress resulted to be facilitators of searching for health-information online and for making treatment decisions.^22,34,37,38,41,44,53^ For example, in Fleisher and colleagues’ study (2002),^38^ participants reported feeling empowered by having easy access to information and not finding it confusing or overwhelming. Moreover, the need to be in control of all the decisions regarding general health and treatments,^33^ together with having anxiety and emotional or informational needs, represented notable facilitators.^33,34,36,37,51,54^ For example, in Yli-Uotila and colleagues’ study (2013),^54^ breast cancer patients expressed the need for emotional support as a result of negative feelings and lack of peer support, which encouraged them to look for further online information and support. Moreover, their needs for informational support were not adequately met by public healthcare systems, which, in turn, resulted in increased online information seeking. Consistently, another prevalent facilitator in the included studies was the presence of social support systems. Indeed, being married, and having a partner, family and friends were detected as facilitators of online information seeking.^36,38,48,50–52^ Colorectal cancer patients participating in one included study reported searching for information online due to experiencing embarrassing symptoms (e.g., digestive and different elimination symptoms) and being ashamed to talk about such symptoms with someone else.^45^ Lastly, post-traumatic stress symptoms, such as Internet rumination and consequent post-traumatic growth (e.g., personal strengths, appreciation of life and spirituality) were also cited as facilitators in patients with breast cancer.^44,49^

*Accessibility.* Living in urban areas^22,34,52,53^ and having broadband Internet access, with the possibility to access the web from multiple devices,^34,41,42,54^ represented important facilitators for cancer patients.

*Quality and quantity of information.* In relation to the quality of the health-related information available online, cancer patients in four studies had the perception that the Internet was useful for health-related information and social support.^35,37,42,44^ For example, prostate cancer reported using eHealth to read/listen to other men's prostate cancer stories, to offer their own and to get personal opinions for help in making a treatment decision.^44^

*Cancer stage and symptoms.* Lower cancer stage and having just been diagnosed were detected as facilitators.^22,36,38,51^ Having a comorbid condition (e.g., hypertension, diabetes, cardiovascular disease or other chronic illnesses) or undergoing aggressive treatments, were reported as facilitators in patients with breast, colorectal and urological cancer.^47,53^ Such patients often searched the Internet for information regarding risks, consequences and benefits of treatment in the short and long term.

*Aspects related to healthcare professionals.* The concern with knowledge limitation of the health care professionals (e.g., nurses, doctors, physicians and clinical centre's staff) or not having access to a healthcare professional were observed as facilitators to Internet search in four studies.^24,37,40,54^ On the other hand, having a regular healthcare professional, good patient task behaviours (e.g., participation with doctors, question asking behaviours, information gathering prior to a doctor appointment and good relationship with doctors^38^) or family members that search for information online were also considered facilitators in three studies.^36,38,42^ Moreover, being referred to trusted websites by healthcare professionals and being guided in learning which information is reliable and up to date or not, were also spotted as facilitators of Internet use.^40^

*Digital literacy.* Good computer skills together with eHealth literacy were found to be facilitators of online information seeking for patients with urological and breast cancer.^24,34^

## Discussion

The current systematic review identified 24 studies that investigated cancer patients’ barriers to and facilitators of online health information-seeking behaviours. Identified themes are coherent with those highlighted in the CMIS,^14^ although reported in slightly different terms. Indeed, we identified both ‘health-related factors’, namely socio-demographics, cancer stage and symptoms and ‘information-carrier characteristics’, namely accessibility and quality and quantity of information, possibly contributing to online information-seeking behaviours. Differently from the model, we did find less information about cognitive evaluations (e.g., beliefs and salience) but we retrieved other potential factors contributing to seeking behaviours such as the role of healthcare professionals and various psychosocial factors (e.g., distress and social support) as well as the seekers’ digital literacy levels.

In line with the existing literature, socio-demographic characteristics were reported both as barriers and facilitators in most of the included studies. Generally, older age, lower income, lower education levels, belonging to a minority ethnicity group and being male were often associated with limited or no engagement in online information-seeking.^2^ While the connection between lower income and reduced online information-seeking behaviour may seem logical due to the costs associated with devices and Internet access, the link with age may be less clear. Specifically, age emerged as a recurring theme across many of the included studies, with 54% of them mentioning older age as both a barrier and a facilitator in the information-seeking process. This finding underscores the ongoing age-related debate in the existing literature on online health information seeking, affirming conflicting viewpoints in previous studies^.^^55,56^ Some studies suggest that age can be a barrier because older individuals may face challenges or resistance to using the Internet due to limited familiarity with technology or physical impairments such as vision problems or motor difficulties^.^^56^ Conversely, recent publications^23,47^ did not identify older age as a barrier to online information seeking, suggesting a potential shift and increase in Internet usage among older adults.^57^

Another relevant result that emerged from the current review is the role of psychosocial aspects on online information-seeking behaviours. It is interesting to note that, equally to age, also psychosocial aspects were reported both as barriers and facilitators (mentioned respectively in 45% and 66% of the included studies). On one hand, coherently with the existing literature,^58^ negative emotions (e.g., anxiety, stress, confusion and need for control) were found to act as defensive mechanisms or as avoidance strategies in various cancer patients for not searching for further online cancer-related information. As suggested by another study,^59^ such negative emotions could discourage cancer patients from seeking information online by negatively affecting their self-efficacy and trust in their health-related and digital skills. On the other hand, having anxiety and a need for control was found to lead cancer patients to search for as much information as possible.^36^ In this case, online health information seeking represents a strategy to cope with negative emotions and feelings.^18^ Perhaps, the latter result can be further supported by the literature on a phenomenon known as ‘cyberchondria’.^60^ Cyberchondria refers to the excessive or repeated online searches for health-related information associated with anxiety-related pathologies and symptoms. Such behaviour has the function of reassuring one's unrealistic health concerns or confirming one's convictions on what is happening to them regarding health conditions, thus promoting the continuation or increase of the searching behaviour as well the anxiety levels, in a vicious circle.^61^ Emerging evidence draws attention to various vulnerability factors that could lead to cyberchondria, including personal characteristics such as female gender, younger age, as well as engagement in particular forms of online behaviour^62^ and these findings are certainly coherent with the factors that emerged from our results.

Regarding social support, the current review highlights the significance of marriage or having a close social network as a facilitator for seeking health-related information online. However, prior studies have presented conflicting findings on the role of social support.^63,64^ It's possible that when a partner, family member or friend expresses concern about a loved one's cancer diagnosis, this concern may extend to the patient, prompting increased online information seeking.^55^ This aligns with the concept of ‘worry’ proposed by Van Stee & Yang (2018)^,^^14^ which identifies cancer-related worry as a catalyst for seeking health information online.

Direct interactions with healthcare professionals were found to be highly significant for cancer patients. The need for face-to-face contact was found to discourage cancer patients to rely on the Internet for cancer-related information.^32^ Cancer patients often report being reassured and supported by direct and open communication with healthcare professionals to the extent that, in many studies, a face to face contact is preferred over online support and information. This confirms the relevance of the doctor–patient relationship, often providing social and emotional support beyond medical advice.^65,66^ However, the current review also detected a certain degree of distrust and concern with the knowledge of the physician, thus contributing to facilitating online information-seeking behaviours.^67^ Moreover, in some studies, healthcare professionals were the ones who discouraged patients from searching for online information. This result is in line with the existing literature, as many healthcare professionals do discourage their patients and the general public from health-related Internet use because the shared information is often inaccurate or promotes unproven treatments.^68^ Nevertheless, there are some patients that reported being referred to trusted websites by healthcare professionals, in fact, there are credible sources that can be addressed if advised by a professional.^69^ The role of the doctor is therefore a strong influencing factor, able to either facilitate or hinder online information-seeking behaviours among cancer patients.

While doctor–patient communication is typically crucial for patients, there are instances in which cancer patients opt to seek online information instead of directly discussing their symptoms related to their colorectal cancer diagnosis with healthcare professionals. This choice may arise from feelings of embarrassment or discomfort in explaining their symptoms^.^^44^ Embarrassment and stigma are actually frequently reported in the literature, not only with respect to colorectal cancer, but also regarding mental health problems,^70^ cervical cancer^71^ and sexually transmitted diseases.^72^ The fact that the patients were ashamed of talking to someone (e.g., family, friends or healthcare professionals) incentivised them to search for information online.

Another relevant and interesting result that emerged from the current review was the effect of Internet accessibility and the digital literacy. Surprisingly, low access to the Internet and low digital skills, whilst apparently considered as crucial factors contributing to online information seeking, were the less frequently mentioned barriers.^73^ Consistent with this perspective, McCloud and colleagues (2016)^74^ observed that despite unrestricted Internet access and no limitations on devices, disparities in online information-seeking behaviour persist. This suggests that accessibility-related barriers might be less significant than commonly assumed.

Aspects related to cancer stage and health condition in general were also found to be correlated with diverse online information-seeking behaviours. For example, a higher cancer stage was detected as a barrier in 12% of the studies, while having just received the cancer diagnosis has shown to be a facilitator (16%).^36,38,51^ These findings emphasise the dynamic nature of cancer patients’ information-seeking behaviours, which can be influenced by their unique circumstances and emotional states.^75^ Perhaps, individuals diagnosed with cancer at an earlier stage may exhibit higher levels of curiosity and a desire for information, as they are at the outset of their journey and seeking guidance on the best course of action. Conversely, those facing more advanced stages of cancer may experience increased anxiety, which can sometimes deter them from actively seeking information online. In fact, some individuals at advanced stages of cancer might become more anxious precisely because of the information they encounter online.^76^ The abundance of information, especially concerning the potential challenges and outcomes associated with advanced cancer, can be overwhelming and distressing. Patients need assistance in navigating the vast sea of online resources, discerning between reliable and misleading information and managing the emotional toll that a cancer diagnosis can bring.

Indeed, strongly connected to the association between cancer stage and seeking behaviours, the quality and quantity of information found on the Internet emerged as barriers to and facilitators of online information-seeking behaviour in 37% of the included studies. Factors related to the information, such as stressfulness and usefulness, reliability, complexity and amount were determinants of the frequency of online information seeking in the included studies and in other literature.^77^ For example, cancer patients were inhibited from searching for information online when they found great amounts of information and did not have the right tools to discern what was reliable and useful for their specific condition. In addition, the information was often difficult to understand.^42,43^ Conversely, perceived usefulness, clarity and self-efficacy motivated the patients to search the Internet for health-related information.^14^

### Study gaps identified

The included studies provided a wide variety of possible barriers to and facilitators of online health-related information-seeking behaviour; however, several gaps in the literature emerged. There is a need to further explore mechanisms that can explain how a specific factor could influence online information seeking in cancer patients. For example, older age was the most frequent barrier; however, processes behind the associations between age and online information-seeking behaviour were not meticulously elaborated in the included studies. Also, to gain a comprehensive understanding of how age influences online health information seeking, further research is needed to include diverse populations and health contexts. Research that encompasses a broader spectrum of age groups, health conditions and socio-demographic backgrounds will help us discern the nuanced factors that impact individuals’ tendencies to seek health information online. Additionally, regarding the cancer stage and symptoms, no explanation or hypothesis was provided on why a higher cancer stage is often associated with absent or lower online information seeking. Moreover, evidence synthesised in the current review highlighted affective (i.e., anxiety) and relational (i.e., social support) components contributing to seeking behaviours. It is important to consider the complex interplay between objective (e.g., socio-demographics and sources), cognitive (e.g., beliefs), affective (e.g., anxiety) and social (e.g., family, friends and healthcare professionals) components.^14^

Therefore, further studies should explore the impact of health conditions, relationship with healthcare professionals and digital literacy on seeking behaviours. The interest in factors influencing online information-seeking behaviour seems to be growing in more recent years,^56^ but the focus appears to be on socio-demographic aspects, which are less likely to be directly modified, instead of accessibility and the role of healthcare professionals. Further research should aim at investigating barriers and facilitators that are modifiable or could be influenced by some kind of intervention such as psychosocial ones. For example, Admiraal and colleagues (2017)^78^ proposed a web-based tailored psycho-educational intervention for breast cancer patients aimed at improving their self-efficacy and control over their condition and observed that the program increased optimism and control in 41% of the patients for a period of 12 weeks longer than for the patients that did not receive the intervention. In addition, further investigations into barriers and facilitators of online information seeking could be of great value when guiding and encouraging the empowerment of the population. Correct and functional Internet use and appropriate digital skills and literacy could enhance general knowledge regarding health, cancer prevention, treatment and support. While many included studies did not extensively address Internet accessibility, it is essential to explore structural and technological solutions to improve Internet infrastructure. A better Internet connection can overcome accessibility barriers in rural or developing regions where poor infrastructure conditions hinder online access.^79^ By understanding the unique challenges faced by underserved populations and implementing targeted interventions to address these challenges, it might be possible to achieve health equity and improving the health outcomes of all individuals, regardless of their race, ethnicity, income or other social determinants of health.

Lastly, further research is needed that focuses on closing the existing digital divide.^80,81^ Actually, it could be relevant to provide certain guidelines to healthcare professionals in order for them to be able to suggest reliable websites to patients who have access to the Internet or provide pamphlets or printed literature to patients who cannot access or use the Internet for various reasons. In addition, healthcare professionals could tailor the intervention and communication to the individual patient in order to enhance one's self-efficacy and confidence in searching for health-related information online.^82,83^

### Limitations and strengths of the current review

The current systematic review presents itself with various limitations related to the search strategies and sources. As mentioned in previous literature,^56^ online information seeking is a complex construct which is related to a wide variety of aspects. Thus, finding relevant keywords that are accurate and specific is quite difficult, hence it is probable that some literature is unfortunately missed. Another plausible limitation could be the criterion of including studies with only adult patients. As younger age was found to be associated with more frequent online information-seeking behaviour, the studies that included minors could have provided maybe a wider or more consistent range of factors related to the health-related Internet search. Moreover, it is important to acknowledge the temporal aspect of the included studies, many of which date back approximately two decades. Although we did not explore whether barriers/facilitators have evolved over time or remained stable, future empirical studies might assess changes in the digital divide and the reasons behind the increased digital literacy of older adults today compared to 20 years ago. Reflecting on the diversity of publication years in the included studies can provide insights into the evolving nature of cancer patients’ digital-seeking behaviours. Lastly, it is important to mention that the included studies were held in a limited geographic area, completely neglecting several countries such as China, Japan, India and others.

The strengths of this review are reflected, first, the inclusion of both qualitative and quantitative studies. Secondly, another strength of the review is the adoption of a theoretical framework (i.e., the CMIS) to guide the review process and systematically synthesise the existing literature on barriers to and facilitators of online information-seeking behaviours. Moreover, most of the included studies were of high quality and used validated and standardised measures. Evidence synthesised in the current review highlights the importance of making Internet use and digital literacy more accessible in the direction of reducing the existing, and relevant, cancer care disparities among cancer patients.^84^ Lastly, the current review entails an additional strength in being pre-registered in PROSPERO beforehand.^85^

## Conclusions

Evidence synthesised in the current review underscores the pressing need to address disparities in Internet access and its use for health information, especially among younger, wealthier and better-educated cancer patients. Digital innovation holds promise for reducing these disparities, but further research is required to bridge the digital divide, improve treatment access, enhance digital literacy, raise awareness and enable early cancer intervention. Notably, limited Internet access often overlaps with difficulties in accessing cancer care, affecting individuals facing challenges like advanced age, rural residence, low income and education levels. However, factors such as social support, psychological aspects and healthcare professional involvement can serve as valuable resources, potentially reducing disparities, even among vulnerable populations. Thus, promoting Internet use for health-related purposes is crucial in reducing disparities in cancer care and information access.

## Supplemental Material

sj-docx-1-dhj-10.1177_20552076231210663 - Supplemental material for Barriers to and facilitators of online health information-seeking behaviours among cancer patients: A systematic review Click here for additional data file. Supplemental material, sj-docx-1-dhj-10.1177_20552076231210663 for Barriers to and facilitators of online health information-seeking behaviours among cancer patients: A systematic review by Giulia Ferraris, Dario Monzani, Veronica Coppini, Lorenzo Conti, Silvia Francesca Maria Pizzoli, Roberto Grasso and Gabriella Pravettoni in DIGITAL HEALTH

sj-docx-2-dhj-10.1177_20552076231210663 - Supplemental material for Barriers to and facilitators of online health information-seeking behaviours among cancer patients: A systematic review Click here for additional data file. Supplemental material, sj-docx-2-dhj-10.1177_20552076231210663 for Barriers to and facilitators of online health information-seeking behaviours among cancer patients: A systematic review by Giulia Ferraris, Dario Monzani, Veronica Coppini, Lorenzo Conti, Silvia Francesca Maria Pizzoli, Roberto Grasso and Gabriella Pravettoni in DIGITAL HEALTH

## References

[1] Vaccarella S Georges D Bray F , et al. Socioeconomic inequalities in cancer mortality between and within countries in Europe: A population-based study. Lancet Reg Health am. - Europe 2023; 25: 100–551. doi:10.1016/j.lanepe.2022.100551 PMC9929598 36818237

[2] Jia X Pang Y Liu LS . Online health information seeking behavior: A systematic review. Healthcare 2021; 9(12): 17–40. doi:10.3390/healthcare9121740 PMC8701665 34946466

[3] Maddock C Camporesi S Lewis I , et al. Online information as a decision making aid for cancer patients: Recommendations from the Eurocancercoms project. Eur J Cancer 2012; 48(7): 1055–1059. doi:10.1016/j.ejca.2011.08.018 22033324

[4] Hesse BW Nelson DE Kreps GL , et al. Trust and sources of health information: the impact of the internet and its implications for health care providers: findings from the first health information national trends survey. Pol Arch Intern Med 2005; 165(22): 2618–2624. doi:10.1001/archinte.165.22.2618 16344419

[5] Pian W Song S Zhang Y . Consumer health information needs: A systematic review of measures. Inf Process & Manag 2020; 57: 102077. doi:10.1016/j.ipm.2019.102077

[6] Shin DS Kim S Jo HS . Understanding facilitators and barriers of online cancer information utilization among cancer survivors and their families: focus on the theory of planned behavior. Asian Pac J Cancer Prev 2020; 21(5): 1357–1362. doi:10.31557/APJCP.2020.21.5.1357 PMC7541892 32458644

[7] Matthews AK Sellergren SA Manfredi C , et al. Factors influencing medical information seeking among african american cancer patients. J Health Commun 2002; 7(3): 205–219. doi:10.1080/10810730290088094 12166874

[8] Bundorf MK Wagner TH Singer SJ , et al. Who searches the internet for health information? Health Serv Res 2006; 41(3 Pt 1): 819–836. doi:10.1111/j.1475-6773.2006.00510.x PMC1713205 16704514

[9] Yang S Lee C Beak J . Social disparities in online health-related activities and social support: findings from health information national trends survey. J Health Commun 2021; 38(7): 1293–1304. doi:10.1080/10410236.2021.2004698 34865570

[10] Morgan PD Fogel J Rose L , et al. African American couples merging strengths to successfully cope with breast cancer. Oncol Nurs Forum 2005; 32(5): 979–987. doi:10.1188/05.ONF.979-987 16136196

[11] Costas-Muniz R Sen R Leng J , et al. Cancer stage knowledge and desire for information: mismatch in Latino cancer patients? J Cancer Educ 2013; 28(3): 458–465. doi:10.1007/s13187-013-0487-8 PMC3755090 23740509

[12] Gustafson DH McTavish FM Stengle W , et al. Use and impact of ehealth system by low-income women with breast cancer. J Health Commun 2005; 10(1): 195–218. doi:10.1080/10810730500263257 16377608

[13] Rice RE . Influences, usage, and outcomes of Internet health information searching: Multivariate results from the Pew surveys. Int J Med Inform 2006; 75(1): 8–28. doi:10.1016/j.ijmedinf.2005.07.032 16125453

[14] Van Stee SK Yang Q . Online cancer information seeking: Applying and extending the comprehensive model of information seeking. J Health Commun 2018; 33(12): 1583–1592. doi:10.1080/10410236.2017.1384350 29083231

[15] Ayers SL Kronenfeld JJ . Chronic illness and health-seeking information on the Internet. Health 2007; 11(3): 327–347. doi:10.1177/1363459307077547 17606698

[16] Van Weert JCM Bolle S Muusses LD . Age and age-related differences in internet usage of cancer patients. In: Stephanidis C and Antona M (eds) *Universal Access in Human-Computer Interaction. Aging and Assistive Environments*. Crete, Greece: Springer Intern Pub, 2014.

[17] Renahy E Parizot I Chauvin P . Determinants of the frequency of online health information seeking: Results of a Web-based survey conducted in France in 2007. Inform Health Soc Care 2010; 35(1): 25–39. doi:10.3109/17538150903358784 PMC3034225 20302437

[18] De Looper M Van Weert JCM Schouten BC , et al. The influence of online health information seeking before a consultation on anxiety, satisfaction, and information recall, mediated by patient participation: Field study. Int J Med Res 2021; 23(7): e23670. doi:10.2196/23670 PMC8290326 34255657

[19] Li N Orrange S Kravitz RL , et al. Reasons for and predictors of patients’ online health information seeking following a medical appointment. Fam Pract 2014; 31(5): 550–556. doi:10.1093/fampra/cmu034 24963151

[20] Hou J Shim M . The role of provider–patient communication and trust in online sources in internet use for health-related activities. J Health Commun 2010; 15(3): 186–199. doi:10.1080/10810730.2010.522691 21154093

[21] Van Dijk J Hacker K . The digital divide as a complex and dynamic phenomenon. Inf Soc 2003; 19(4): 315–326. doi:10.1080/01972240309487

[22] Chou WY Liu B Post S , et al. Health-related Internet use among cancer survivors: data from the Health Information National Trends Survey, 2003-2008. J Cancer Surviv: research and practice 2011; 5(3): 263–270. doi:10.1007/s11764-011-0179-5 21505861

[23] Jiang S Liu PL . Digital divide and Internet health information seeking among cancer survivors: A trend analysis from 2011 to 2017. Psychooncol 2020b; 29(1): 61–67. doi:10.1002/pon.5247 31652360

[24] Duimel SLL Linn AJ Smets EMA , et al. Profiling cancer patients based on their motives for seeking informational and emotional support online. J Health Commun 2022; 22: 1–15. doi:10.1080/10410236.2022.2144287 36415021

[25] Lleras De Frutos M Casellas-Grau A Sumalla EC , et al. A systematic and comprehensive review of internet use in cancer patients: Psychological factors. Psychooncol 2020; 29(1): 6–16. doi:10.1002/pon.5194 31385400

[26] Page MJ McKenzie JE Bossuyt PM , et al. The PRISMA 2020 statement: An updated guideline for reporting systematic reviews. Br Med J 2021; 372: 71. doi:10.1136/bmj.n71 PMC8005924 33782057

[27] Methley AM Campbell S Chew-Graham C , et al. PICO, PICOS and SPIDER: A comparison study of specificity and sensitivity in three search tools for qualitative systematic reviews. BMC Health Serv Res 2014; 14(1): 579. doi:10.1186/s12913-014-0579-0 PMC4310146 25413154

[28] Ouzzani M Hammady H Fedorowicz Z , et al. Rayyan—A web and mobile app for systematic reviews. Syst Rev 2016. doi:10.1186/s13643-016-0384-4 PMC5139140 27919275

[29] Belur J Tompson L Thornton A , et al. Interrater reliability in systematic review methodology: Exploring Variation in coder decision-making. Sociol Method Res 2021; 50(2): 837–865. doi:10.1177/0049124118799372

[30] Hong QN Pluye P Fàbregues S , et al. Mixed Methods Appraisal Tool (MMAT), Version 2018. User guide. Montreal, MTL: McGill, 2018; 34(4): 285–291. doi:10.3233/EFI-180221

[31] Thomas J Harden A . Methods for the thematic synthesis of qualitative research in systematic reviews. BMC Med Res Method 2008; 8: 45. doi:10.1186/1471-2288-8-45 PMC2478656 18616818

[32] Lizarondo L Stern C Carrier J , et al. Mixed methods systematic reviews. Chapter 8: mixed methods systematic reviews. In: Aromataris E Munn Z (eds) JBI Manual for Evidence Synthesis. Adelaide, Australia: JBI, 2020. Available from https://synthesismanual.jbi.global. doi:10.46658/JBIMES-20-09

[33] Balka E Krueger G Holmes BJ , et al. situating internet use: Information-seeking among young women with breast cancer. J Comput Mediat Commun 2010; 15(3): 389–411. doi:10.1111/j.1083-6101.2010.01506.x

[34] Bender JL Feldman-Stewart D Tong C , et al. Health-related internet use among men with prostate cancer in canada: cancer registry survey study. J Med Intern Res 2019; 21(11): e14241. doi:10.2196/14241 PMC6891399 31742561

[35] An LC Wallner L Kirch MA . online social engagement by cancer patients: A clinic-based patient survey. J Med Intern R 2016; 2(2): e10. doi:10.2196/cancer.5785 PMC5369628 28410186

[36] Corrales DM Wells AE Radecki Breitkopf C , et al. Internet use by gynecologic oncology patients and its relationship with anxiety. J Health Commun 2018; 23(3): 299–305. doi:10.1080/10810730.2018.1442529 29474124

[37] Dickerson SS Reinhart A Boemhke M , et al. Cancer as a problem to be solved: Internet use and provider communication by men with cancer. Comput Inform Nurs 2011; 29(7): 388–395. doi:10.1097/NCN.0b013e3181f9ddb1 20975535

[38] Fleisher L Bass SB McKeown-Conn N . Relationships among internet health information use, patient behavior and self-efficacy in newly diagnosed cancer patients who contact the national cancer institute’s (NCI) atlantic region cancer information service (CIS). Proceedings. AMIA Symposium 2002: 260–264. 12463827 PMC2244151

[39] George GC Buford A Hess K , et al. Cancer-Related Internet Use and Online Social Networking Among Patients in an Early-Phase Clinical Trials Clinic at a Comprehensive Cancer Center. JCO Clin Cancer Inform 2018; 2: 1–14. doi:10.1200/CCI.17.00030 PMC6874009 30652565

[40] Haase KR Sattar S Holtslander L , et al. The role of internet cancer information for older adults with cancer: Perspectives of older adults and healthcare professionals. Int J Older People Nurs 2020, 15(2): e12303. doi:10.1111/opn.12303 31922334

[41] Jiang S Liu J . Examining the relationship between Internet health information seeking and patient-centered communication in China: Taking into account self-efficacy in medical decision-making. Chin J Commun 2020a; 13(1): 1–18. doi:10.1080/17544750.2020.1769700

[42] Mayer DK Terrin NC Kreps GL , et al. Cancer survivors information seeking behaviors: A comparison of survivors who do and do not seek information about cancer. Patient Educ Couns 2007; 65(3):342–350. doi:10.1016/j.pec.2006.08.015 PMC5693234 17029864

[43] Peterson MW Fretz PC . Patient use of the internet for information in a lung cancer clinic*. Chest 2003; 123(2):452–457. doi:10.1378/chest.123.2.452 12576365

[44] Rising CJ Bol N Kreps GL . Age-related use and perceptions of eHealth in men with prostate cancer: A web-based survey. JMIR cancer 2015; 1(1): 6. doi:10.2196/cancer.4178 PMC5367670 28410165

[45] Thomson MD Siminoff LA Longo DR . Internet use for prediagnosis symptom appraisal by colorectal cancer patients. Health Educ Behav 2012; 21(2): 12–400. doi:10.1177/1090198111423941 PMC3521844 21990571

[46] Tian Y Robinson JD . Incidental health information use and media complementarity: a comparison of senior and non-senior cancer patients. Patient Educ Couns 2008; 71(3): 340–344. doi:10.1016/j.pec.2008.02.006 18372141

[47] Melhem SJ Nabhani-Gebara S Kayyali R . Digital trends, digital literacy, and e-health engagement predictors of breast and colorectal cancer survivors: A population-based cross-sectional survey. Int J Environ Res Public Health 2023; 30(8): 6827–6837. doi:10.3390/ijerph20021472 PMC9860554 36674237

[48] Paul CL Carey ML Hall AE , et al. Improving access to information and support for patients with less common cancers: hematologic cancer patients’ views about Web-based approaches. JMIR 2011; 13(4): 112. doi:10.2196/jmir.1894 PMC3278098 22189354

[49] Casellas-Grau A Sumalla EC Lleras M , et al. The role of posttraumatic stress and posttraumatic growth on online information use in breast cancer survivors. Psychooncol 2018; 27(8): 1971–1978. doi:10.1002/pon.4753 29740909

[50] Kowalski C Kahana E Kuhr K , et al. Changes over time in the utilization of disease-related Internet information in newly diagnosed breast cancer patients 2007 to 2013. JMIR 2014; 16(8): 195. doi:10.2196/jmir.3289 PMC4180359 25158744

[51] Mattsson S Olsson EMG Johansson B , et al. Health-related internet use in people with cancer: Results from a cross-sectional study in two outpatient clinics in Sweden. JMIR 2017; 19(5): 163. doi:10.2196/jmir.6830 PMC5447824 28506959

[52] Valero-Aguilera B Bermúdez-Tamayo C García-Gutiérrez JF , et al. Factors related to use of the Internet as a source of health information by urological cancer patients. Support Care Cancer 2012; 20(12): 3087–3094. doi:10.1007/s00520-012-1431-x 22415609

[53] Valero-Aguilera B Bermúdez-Tamayo C García-Gutiérrez JF , et al. Information needs and Internet use in urological and breast cancer patients. Support Care Cancer 2014; 22(2): 545–552. doi:10.1007/s00520-013-2009-y 24122406

[54] Yli-Uotila T Rantanen A Suominen T . Motives of cancer patients for using the Internet to seek social support. Eur J Cancer Care 2013; 22(2): 261–271. doi:10.1111/ecc.12025 23320398

[55] Pourrazavi S Hashemiparast M Bazargan-Hejazi S , et al. Why older people seek health information online: A qualitative study. Adv Gerontol 2021; 11(3): 290–297. doi:10.1134/S2079057021030115

[56] Zhao YC Zhao M Song S . Online health information seeking behaviors among older adults. Systematic Scoping Review. JMIR 2022; 24(2): e34790. doi:10.2196/34790 PMC8892316 35171099

[57] Yoon H Jang Y Kim S , et al. Trends in internet use among older adults in the United States, 2011–2016. J Appl Gerontol 2021; 40(5): 466–470. doi:10.1177/0733464820908427 32131670

[58] Baumgartner SE Hartmann T . the role of health anxiety in online health information search. Cyberpsychol Behav, Soc Netw 2011; 14(10): 613–618. doi:10.1089/cyber.2010.0425 21548797

[59] Linn AJ Van Weert JCM Gebeyehu BG , et al. Patients’ online information-seeking behavior throughout treatment: The impact on medication beliefs and medication adherence. J Health Commun 2019; 34(12): 1461–1468. doi:10.1080/10410236.2018.1500430 30052088

[60] Schenkel SK Jungmann SM Gropalis M , et al. Conceptualizations of cyberchondria and relations to the anxiety spectrum. Systematic Review and Meta-analysis. JMIR 2021; 23(11): e27835. doi:10.2196/27835 PMC8663695 34792473

[61] McMullan RD Berle D Arnáez S , et al. The relationships between health anxiety, online health information seeking, and cyberchondria: Systematic review and meta-analysis. J Affect Disord 2019; 245: 270–278. doi:10.1016/j.jad.2018.11.037 30419526

[62] Vismara M Varinelli A Pellegrini L , et al. New challenges in facing cyberchondria during the coronavirus disease pandemic. Curr Opin Behav Sci 2022; 46: 101–156. doi:10.1016/j.cobeha.2022.101156 PMC9098916 35581995

[63] Kim SC Shah DV Namkoong K , et al. Predictors of online health information seeking among women with breast cancer: The role of social support perception and emotional well-being: social support, emotional well-being, and online information seeking. J Comput Mediat Commun 2013; 18(2): 98–118. doi:10.1111/jcc4.12002 PMC3951120 24634575

[64] Nambisan P . Information seeking and social support in online health communities: Impact on patients’ perceived empathy. J Am Med Inform Assoc 2011; 18(3): 298–304. doi:10.1136/amiajnl-2010-000058 PMC3078657 21486888

[65] Baumeister RF Leary MR . The need to belong: desire for interpersonal attachments as a fundamental human motivation. Psychol Bull 1995; 117: 497–529. doi:10.1037/0033-2909.117.3.497 7777651

[66] Newson M Zhao Y Zein ME , et al. Digital contact does not promote wellbeing, but face-to-face contact does: A cross-national survey during the COVID-19 pandemic. New Media Soc 2021; 00(0): 1–24. doi:10.1177/14614448211062164 PMC10758341 38174349

[67] Luo A Qin L Yuan Y , et al. The effect of online health information seeking on physician-patient relationships. Systematic Review. JMIR 2022; 24(2): e23354. doi:10.2196/23354 PMC8874798 35142620

[68] Johnson SB Parsons M Dorff T , et al. Cancer misinformation and harmful information on facebook and other social media: A brief report. JNCI 2022; 114(7): 1036–1039. doi:10.1093/jnci/djab141 PMC9275772 34291289

[69] National Cancer Institute. Addressing the Challenges of Cancer Misinformation on Social Media. NCI. 2022.https://www.cancer.gov/news-events/cancer-currents-blog/2021/cancer-misinformation-social-media. Accessed Mar 2023.

[70] Pretorius C Chambers D Coyle D . Young people’s online help-seeking and mental health difficulties. Systematic Narrative Review. JMIR 2019; 21(11): e13873. doi:10.2196/13873 PMC6891826 31742562

[71] Williams RC Simonds H Roomaney R . Knowledge, misinformation, stigma, and disclosure hesitancy among women receiving curative treatment for cervical cancer at a tertiary hospital in South Africa. S Afr J Psychol 2023; 53(3): 315–326. doi:10.1177/00812463221148323

[72] Neal TMS Lichtenstein B Brodsky SL . Clinical implications of stigma in HIV/AIDS and other sexually transmitted infections. Int J STD AIDS 2010; 21(3): 158–160. doi:10.1258/ijsa.2008.008445 20215618

[73] Lee HY Jin SW Henning-Smith C , et al. Role of health literacy in health-related information-seeking behavior online: Cross-sectional study. JMIR 2021; 23(1): 14–88. doi:10.2196/14088 PMC7875696 33502332

[74] McCloud RF Okechukwu CA Sorensen G , et al. Beyond access: barriers to internet health information seeking among the urban poor. J Am Med Inform Assoc 2016; 23(6): 1053–1059. doi:10.1093/jamia/ocv204 PMC5070515 27206459

[75] National Cancer Institute. Crunching Numbers: What Cancer Screening Statistics Really Tell Us. NCI. 2018. https://www.cancer.gov/about-cancer/screening/research/what-screening-statistics-mean. Accessed Mar 2023.

[76] Perrault EK Hildenbrand GM McCullock SP , et al. Online information seeking behaviors of breast cancer patients before and after diagnosis: From website discovery to improving website information. Cancer Treat Res Commun 2020; 23: 100–176. doi:10.1016/j.ctarc.2020.100176 32388484

[77] Wang, Xiu, & Shahzad. Exploring the determinants of online health information-seeking behavior using a meta-analytic approach. Sustain Sci 2019; 11(17): 1–14. doi:10.3390/su11174604

[78] Admiraal JM van der Velden AWG Geerling JI , et al. Web-based tailored psychoeducation for breast cancer patients at the onset of the survivorship phase: A multicenter randomized controlled trial. J Pain Symptom Manage 2017; 54(4): 466–475. doi:10.1016/j.jpainsymman.2017.07.009 28711750

[79] Sharma R Mokhtar IA . Bridging the digital divide in Asia. Int J Technol Knowl Soc 2006; 1(3): 15–30. doi:10.18848/1832-3669/CGP/v01i03/55886

[80] Kind T Wallace J Moon RY . The digital divide: A comparison of online consumer health information for african-american and general audiences. J Natl Med Assoc 2008; 100(11): 1333–1340. doi:10.1016/S0027-9684(15)31513-3 19024231

[81] Wagner TH Bundorf MK Singer SJ , et al. Free internet access, the digital divide, and health information. Med Care 2005; 43(4): 415–420. doi:10.1097/01.mlr.0000156857.14152.6e 15778645

[82] Epstein RM Duberstein PR Fenton JJ , et al. Effect of a patient-centered communication intervention on oncologist-patient communication, quality of life, and health care utilization in advanced cancer: The VOICE randomized clinical trial. JAMA Oncol 2016; 3(1): 92–100. doi:10.1001/jamaoncol.2016.4373 PMC5832439 27612178

[83] Niu Z Willoughby J Zhou R . Associations of health literacy, social media use, and self-efficacy with health information–seeking intentions among social media users in China: Cross-sectional survey. JMIR 2021; 23(2): e19134. doi:10.2196/19134 PMC7952238 33629955

[84] Bayard S Fasano G Gillot T , et al. Breast cancer disparities and the digital divide. Curr Breast Cancer Rep 2022; 14(4): 205–212. doi:10.1007/s12609-022-00468-w PMC9703401 36467667

[85] Ioannidis JPA . How to make more published research true. PLoS Med 2014; 11(10): e1001747. doi:10.1371/journal.pmed.1001747 PMC4204808 25334033

