# Supplementary material for: Barriers to and facilitators of online health information-seeking behaviours among cancer patients: A systematic review
Source: Digit Health. 2023 Dec 15;9:20552076231210663. doi: 10.1177/20552076231210663 (PMC10725105; doi:10.1177/20552076231210663)
Supplement: sj-docx-2-dhj-10.1177_20552076231210663 - Supplemental material for Barriers to and facilitators of online health information-seeking behaviours among cancer patients: A systematic review [file sj-docx-2-dhj-10.1177_20552076231210663.docx]

Supplementary material 2

**Search strategy**

| **Database** | **Search string** | **N° of studies** |
| --- | --- | --- |
| **PUBMED** | ( ((("Neoplasms"[Mesh] OR neoplasm OR neoplastic OR cancer OR tumor OR tumour OR oncology OR Oncological* OR malignan* OR metastasis OR metastases OR metastat*) AND ("Communication Barriers"[Mesh] OR barrier[tw] OR facilitator[tw] OR "digital divide"[tw] OR "digital gap" OR "digital inequalit*" OR "digital inclusion" OR "Attitude to Health"[Mesh] OR "Attitude"[Mesh] OR "health attitude"[TW] OR "health beliefs"[TW] OR "health perception*"[TW] OR "fear"[MeSH Terms] OR "fear"[TW] OR "fearful"[TW] OR "fearfulness"[TW] OR "anxiety"[MeSH Terms] OR "anxiety"[TW] OR "anxious"[TW] OR "shame"[MeSH Terms] OR "shame"[TW] OR ("Social Support"[Mesh]) OR "Psychosocial Support Systems"[Mesh] OR "social support"[TW] OR "emotional well being"[TW] OR "emotional wellbeing"[TW] OR "Affective Symptoms"[Mesh] OR "affective symptom*"[TW] OR "emotional response*"[TW] OR "emotional disturbance*"[TW] OR psycholog*[TI] OR "psychosocial*"[TI] OR "psycho-social*"[TI])) AND (((("Information Seeking Behavior"[Mesh]) OR "Health Information Exchange"[Mesh]) OR "Access to Information"[Mesh]) OR "Consumer Health Information"[Mesh] OR "Health Literacy"[Mesh] OR "information seeking" OR "health information" OR "information literacy" OR "ehealth literacy" OR "e-health literacy" OR "Health literacy" OR telemedicine)) AND ("Internet Access"[Mesh] OR "Internet Use"[Mesh] OR "Internet"[Mesh] OR online[TW] OR internet[TW] OR “social media”[TW] OR web[TI])) | **917** |
| **SCOPUS** | ((TITLE-ABS-KEY ("Communication Barriers" ) OR TITLE-ABS-KEY ( barrier ) OR TITLE-ABS-KEY ( facilitator ) OR TITLE-ABS-KEY ( "digital divide" ) OR "digital gap" OR "digital inequalit*" OR "digital inclusion" OR TITLE-ABS-KEY ( "Attitude to Health" ) OR TITLE-ABS-KEY ( attitude ) OR TITLE-ABS-KEY ( "health attitude" ) OR TITLE-ABS-KEY ( "health beliefs" ) OR TITLE-ABS-KEY ( "health perception*" ) OR TITLE-ABS-KEY ( fear ) OR TITLE-ABS-KEY ( fear ) OR TITLE-ABS-KEY ( fearful ) OR TITLE-ABS-KEY ( fearfulness ) OR TITLE-ABS-KEY ( anxiety ) OR TITLE-ABS-KEY ( anxiety ) OR TITLE-ABS-KEY ( anxious ) OR TITLE-ABS-KEY ( shame ) OR TITLE-ABS-KEY ( shame ) OR ( TITLE-ABS-KEY ( "Social Support" ) ) OR TITLE-ABS-KEY ( "Psychosocial Support" ) OR TITLE-ABS-KEY ( "social support" ) OR TITLE-ABS-KEY ( "emotional well being" ) OR TITLE-ABS-KEY ( "emotional wellbeing" ) OR TITLE-ABS-KEY ( "Affective Symptoms" ) OR TITLE-ABS-KEY ( "affective symptom*" ) OR TITLE-ABS-KEY ( "emotional response*" ) OR TITLE-ABS-KEY ( "emotional disturbance*" ) OR TITLE-ABS-KEY ( psycholog* ) OR TITLE-ABS-KEY ( psychosocial* ) OR TITLE-ABS-KEY ( psycho-social* )) AND (TITLE-ABS-KEY(cancer OR tumor OR tumour OR oncolog* OR malignan* OR neoplas* OR metasta*))) AND (TITLE-ABS-KEY ("Information Seeking") OR TITLE-ABS-KEY ("Health Information") OR TITLE-ABS-KEY ("Access to Information") OR TITLE-ABS-KEY( "health information") OR TITLE-ABS-KEY("information literacy") OR TITLE-ABS-KEY("ehealth literacy") OR TITLE-ABS-KEY ("e-health literacy") OR TITLE-ABS-KEY ("Health literacy") OR TITLE-ABS-KEY(telemedicine)) AND NOT INDEX(medline) | **871** |
| **EMBASE** | ('communication barrier'/exp OR 'communication barrier' OR barrier* OR facilitator* OR 'digital divide'/exp OR 'digital divide' OR 'digital gap' OR 'digital inequalit*' OR 'digital inclus*' OR 'attitude to health'/exp OR 'attitude to health' OR 'health attitude*' OR 'health belief*' OR 'fear' OR 'fear'/exp OR fear OR 'anxiety' OR 'anxiety'/exp OR anxiety OR 'shame' OR 'shame'/exp OR shame OR 'psychosocial care'/exp OR 'psychosocial care' OR 'psychosocial support'/exp OR 'psychosocial support' OR 'social support'/exp OR 'social support' OR 'emotional well-being'/exp OR 'emotional well-being' OR 'emotional wellbeing'/exp OR 'emotional wellbeing' OR 'emotional disorder'/exp OR 'emotional disorder' OR 'affective symptom*') AND ('malignant neoplasm'/exp OR 'malignant neoplasm' OR 'neoplasm' OR 'neoplasm'/exp OR neoplasm OR 'cancer patient'/exp OR 'cancer patient' OR neoplastic OR 'cancer' OR 'cancer'/exp OR cancer OR 'tumor' OR 'tumor'/exp OR tumor OR 'tumour' OR 'tumour'/exp OR tumour OR 'oncology' OR 'oncology'/exp OR oncology OR oncological* OR malignan* OR 'metastasis' OR 'metastasis'/exp OR metastasis OR 'metastases' OR 'metastases'/exp OR metastases OR metastat*) AND ('information seeking'/exp OR 'information seeking' OR 'access to information'/exp OR 'access to information' OR 'health literacy'/exp OR 'health literacy' OR 'information literacy'/exp OR 'information literacy' OR 'ehealth literacy'/exp OR 'ehealth literacy' OR 'e-health literacy'/exp OR 'e-health literacy') AND ('internet access'/exp OR 'internet access' OR 'internet use'/exp OR 'internet use' OR 'internet' OR 'internet'/exp OR internet OR 'social media'/exp OR 'social media' OR web:ti OR internet:ti OR online:ti) | **591** |
